# Development and validation of a 13-item short version of the inflammatory bowel disease self-efficacy scale

**DOI:** 10.1186/s12876-024-03206-x

**Published:** 2024-05-31

**Authors:** Makoto Tanaka, Aki Kawakami, Kayoko Sakagami, Tomoko Terai, Jovelle Fernandez, Laurie Keefer, Hiroaki Ito

**Affiliations:** 1 Graduate School of Health Care Sciences, Tokyo Medical and Dental University (TMDU), Tokyo, Japan; 2 Kinshukai Infusion Clinic, Osaka, Japan; 3 Japan Medical Office, Takeda Pharmaceutical Company Limited, Tokyo, Japan; 4 Icahn School of Medicine at Mount Sinai, New York City, NY USA; 5 Present Address: L.L.C., Middletown, DE USA

**Keywords:** Inflammatory bowel disease, Patient-reported outcome measures, Self-efficacy

## Abstract

**Background:**

The inflammatory bowel disease self-efficacy scale (IBD-SES) is an instrument used across many countries to measure important health outcomes of patients with inflammatory bowel disease (IBD). We aimed to develop and validate a substantially shorter version of this scale to reduce patients’ response burden.

**Methods:**

A total of 919 patients with IBD, 482 recruited from an IBD clinic and 437 recruited online, completed the Japanese version of the original, 29-item IBD-SES. These data were then used to develop a shorter version of the scale. The original 29 items of the IBD-SES were reduced with three analytic steps: assessing ceiling and floor effect, testing correlation between items, and assessing test-retest reliability. The resulting 13-item IBD-SES was evaluated for construct validity by confirmatory factor analysis, criterion validity by Pearson correlation coefficients with original version, and internal consistency by item-total correlations and the Cronbach’s α coefficient.

**Results:**

The short version consisted of the same four subscales “managing stress and emotions,” “managing medical care,” “managing symptoms and disease,” and “maintaining remission” as the original scale. The fit indices of the final model were as follows: normed chi-square, 7.18 (*p* < 0.001); comparative fit index, 0.94; goodness**-**of**-**fit index, 0.93; adjusted goodness-of-fit index, 0.89; parsimony goodness-of-fit index, 0.60; and root mean square error of approximation, 0.084. Correlation of each subscale with the original scale was high (0.97–0.98). Cronbach’s α for each subscale ranged from 0.68 to 0.86.

**Conclusions:**

A short version of the IBD-SES was developed. The results confirmed the improved validity, reliability, and psychometric properties of the IBD-SES.

**Trial registration:**

Not applicable.

**Supplementary Information:**

The online version contains supplementary material available at 10.1186/s12876-024-03206-x.

## Background

Patients with inflammatory bowel disease (IBD), including Crohn’s disease (CD) and ulcerative colitis (UC), face difficulty in their social life and are required to self-manage their disease and cope with their condition. Patients with IBD sometimes fail to adhere to their required health management, such as maintaining medication adherence, adjusting daily life based on self-monitoring and appropriate clinical visits [1]. According to a previous review and established guidelines, poor self-management is often associated with poorer mental and physical health. Conversely, effective self-management is associated with reduced symptoms, fewer hospitalizations, and reduced need for long-term treatment [1, 2]. Health professionals should play important roles in rendering self-management education, empowering patients, and supporting them to better manage their disease. Assessing and promoting patient self-efficacy is recommended to provide effective and efficient support for patients in self-managing their illness [1]. 

Self-efficacy is defined as “the conviction that one can successfully execute the behavior required to produce the outcomes” [3] and is one of the key concepts for successful self-management. Self-efficacy is a central concept in self-management education [4] with numerous studies demonstrating its correlation with optimal health behaviors for the self-management in patients with chronic diseases [5–7]. In chronic diseases like IBD, self-efficacy has been proven to be one of the important health outcomes [8]. Recognizing patients’ vulnerable areas by assessing their self-efficacy in managing IBD could pave the way for providing support and fortifying these specific aspects. To assess patients’ self-efficacy in managing various self-management tasks related to IBD, several scales have been developed [9–13]. The IBD self-efficacy scale (IBD-SES) is a measurement tool used widely across many countries [9, 12, 14–17] with psychometric properties that predict psychological distress, showing moderate correlation with quality of life [9]. Although the 29-item IBD-SES is useful, a shorter instrument would increase the likelihood of usage, because survey length can affect response rate [18, 19]. Furthermore, the development of a shortened scale is crucial for optimizing data collection, thereby saving time and reducing respondent burden, particularly in research and clinical settings where practical constraints are substantial. Therefore, in this study, we aimed to develop a substantially shorter, but still valid, version of the IBD-SES. The intention of developing a short version of the IBD-SES was not only to select items that have proper psychometric properties, but also to determine the important aspects of the original scale.

## Methods

### Study design and data collection

We developed the short version of the IBD-SES by reanalyzing data collected in a study designed to validate the Japanese version of the IBD-SES in patients with IBD in Japan [17]. Drawing on guidebooks for scale development [20–22] and referencing published articles on the development of shorter versions of existing scales [23, 24], we formulated a methodology incorporating item reduction and a comprehensive psychometric evaluation. The original, 29-item IBD-SES is a 10-point Likert scale which score ratings from 1 (not at all) to 10 (totally) for each item, reflecting a 2-week timeframe, with the following four subscales: (1) managing stress and emotions, (2) managing medical care, (3) managing symptoms and disease, and (4) maintaining remission [9]. This instrument with higher scores indicates greater self-efficacy.

In this study, cross-sectional questionnaires were distributed to the participants recruited from two sources. The initial survey was conducted at a specialized IBD clinic between July and September 2019. During this period, 500 patients with IBD were consecutively recruited, and only 482 patients actively participated in the study. Information was acquired through a self-administered questionnaire and review of medical care records. Completed questionnaires were collected onsite or via postal mail. All patients were asked to repeat the IBD-SES two weeks after the initial survey to assess test-retest reliability. The second survey was conducted between June and July 2020 using a patient panel managed by QLife Inc. (Tokyo, Japan). Online group recruitment concluded when the target number of applicants was nearly attained on a first-come-first-serve basis. A total of 437 valid responses from 493 participants were analyzed. The details of the survey are described in our previous paper [17]. 

### Item reduction

**Fig. 1:**
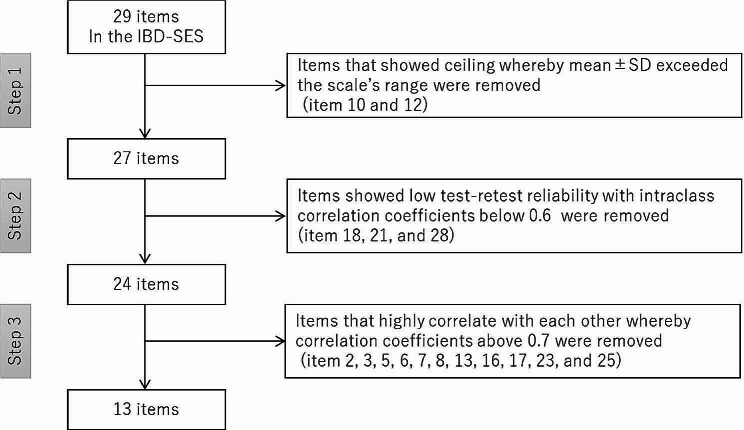
Flow chart showing the development of the short version of the IBD-SES Three analytic steps were used to reduce the number of items from 29 to 13 IBD-SES, the original, 29-item version of the inflammatory bowel disease self-efficacy scale; SD: standard deviation

To maintain the factor structure of the original scale and focus on the crucial components of the factors, the original, 29-item IBD-SES was reduced in three major analytic steps (Fig. 1). The first step was to evaluate the distribution of scores for each item. If the mean ± standard deviation (SD) of the scores for an item exceeded 10 or was below 10 on the scale, it was regarded as a ceiling or floor effect, respectively, and the item was removed. The exclusion of items exhibiting ceiling or floor effects would increase the sensitivity to change, contributing to the overall validity of the assessment. The next step was to assess test-retest reliability by examining intraclass correlation coefficients (ICCs) (2,1) between IBD-SES scores across a two-week interval in participants from the clinic. Items with ICCs below 0.6 were removed. Following the elimination of items exhibiting diminished reliability or validity, the third step was to use the Pearson correlation coefficient to explore the association between items. If pairs of items displayed a high correlation (|r| ≥ 0.7), exclusion was considered to minimize redundancy. Evaluation and selection of items representing the main aspects of the subscales were conducted through expert group discussions. The first (M.T.) and second (A.K.) authors discussed and identified the main aspects of each subscale, and formulated items for potential selection. To ensure reliability and adhere to the factor analysis considerations, a minimum of three items were included in each subscale. Subsequently, an online meeting (involving M.T., A.K., K.S., T.T., and H.I.) was held to reach a consensus. L.K. agreed to the draft reported through e-mail.

### Psychometric evaluation of the short version of the IBD-SES

The short version of the scale was evaluated for reliability and validity. For reliability, in addition to the previously mentioned ICCs, internal consistency was assessed by calculating item-total correlations and the Cronbach’s α coefficient. Construct validity was evaluated by confirmatory factor analysis and criterion validity was evaluated with Pearson correlation coefficients between subscales in the short version and the original version. We hypothesized that the short version has the same four subscales as the original version: managing stress and emotions, managing medical care, managing symptoms and disease, and maintaining remission. The model fit was assessed with the comparative fit index (CFI), goodness-of-fit index (GFI), adjusted goodness-of-fit index (AGFI), parsimony goodness-of-fit index (PGFI), and root mean square error of approximation (RMSEA).

Statistical analyses were performed with IBM SPSS v26.0 J and IBM Amos v26.0 J for Windows. Statistical significance level was set to 0.05.

## Results

A total of 919 valid responses were obtained with 482 (ulcerative colitis: 184, Crohn’s disease: 298) patients from a specialized IBD clinic and 437 (ulcerative colitis: 255, Crohn’s disease: 182) patients recruited online. Table 1 shows the characteristics of participants, more details are shown in our previous paper [17]. 

**Table 1:** Characteristics of patients

Variables	*N* = 919
Age (Mean ± SD [range])	40.9 ± 11.3 (20–86)
Gender: women	390 (42.4)
Marital status: married	503 (54.7)
Diagnosis: CD	480 (52.2)
Currently in remission^a^	534 (58.1)
Current therapy: Biologic	551 (60.0)
Disease duration (year) (Mean ± SD [range])	12.1 ± 8.8 (0–46)

Data are n (%) unless specified otherwise

CD, Crohn’s disease; SD, standard deviation

^a^Definition: clinic sample (partial Mayo score = 0 or Crohn’s Disease Activity Index < 150); online sample (stool frequency = normal, visible bleeding = none, body temperature = normal)

The items selected for the short version on the basis of analyses of the 29 items of the original IBD-SES are shown in Table 2 (see also Fig. 1 and Supplement Table). Two items were excluded because of a ceiling effect: most patients reported that they “follow medication prescription” and “take medication as directed to prevent flare-up”. Three more items were removed because of low ICC. One of the items removed because of low ICC, item 28 (“engage in stress management program”), had the most missing values of any item (1.6% of the participants). Eleven items that had high correlation with other items were removed to eliminate duplication. Items with high correlation to other items were selected to be retained in the short version of the IBD-SES on the basis of our interpretation of the main aspects contained within each subscale of the original IBD-SES (Table 3). Although items 14 and 15 had a correlation coefficient higher than 0.7, both items were retained to keep the number of items in each subscale at three or greater. In this way, the 29-item original IBD-SES was reduced to a 13-item IBD-SES (IBD-SES13).

**Table 2:** Descriptive and psychometric statistics for items and subscales in the short version of the IBD-SES

IBD-SES13 subscales and items		Item No. within IBD-SES	*N*	Item score,^a^ mean (SD)	Subscale score, mean (SD)	Corrected item-total correlation	Test-retest reliability ICC (95% CI) ^b^	Reliability Cronbach’sα	Correlation with IBD-SES^c^
Managing stress and emotions (range: 3–30)			919	4.6 (1.8) ^d^	13.7 (5.5)			0.68	0.98
	Keep from getting stressed	1	919	3.7 (2.2)		0.53	0.68 (0.58–0.75)		
	Do something to reduce discouragement	4	919	4.9 (2.3)		0.67	0.69 (0.63–0.75)		
	Get emotional support	9	919	5.1 (2.5)		0.53	0.74 (0.68–0.79)		
Managing medical care (range: 3–30)			915	7.2 (2.1) ^d^	21.5 (6.3)			0.81	0.97
	Take medication at instructed times	11	916	7.0 (2.6)		0.33	0.69 (0.62–0.75)		
	Ask doctor about illness	14	918	7.3 (2.3)		0.55	0.69 (0.63–0.75)		
	Discuss problems with medications	15	918	7.2 (2.4)		0.56	0.69 (0.62–0.75)		
Managing symptoms and disease (range: 4–40)			906	4.8 (1.9) ^d^	19.4 (7.6)			0.86	0.98
	Keep sleep problems from interfering	19	916	5.4 (2.6)		0.64	0.64 (0.57–0.71)		
	Keep discomfort / pain from interfering	20	917	5.0 (2.2)		0.73	0.62 (0.54–0.69)		
	Keep symptoms from interfering	22	914	4.8 (2.2)		0.74	0.66 (0.59–0.72)		
	Keep fatigue from interfering	24	915	4.3 (2.1)		0.69	0.67 (0.60–0.73)		
Maintaining remission (range: 3–30)			913	5.1 (1.9) ^d^	15.4 (5.7)			0.79	0.97
	Keep disease in remission	26	913	5.3 (2.2)		0.63	0.69 (0.63–0.75)		
	Engage in self-care (exercise, diet, rest)	27	919	5.3 (2.2)		0.68	0.65 (0.58–0.71)		
	Maintain your sense of well-being	29	919	4.9 (2.3)		0.71	0.78 (0.73–0.82)		

^a^Score range for each item is 1 to 10; higher scores reflect a higher level of perceived self-efficacy

^b^Number of analyzed respondents, *n* = 280

^c^Number of analyzed respondents, *n* = 873

^d^Mean (SD) of mean item scores within subscale

IBD-SES, the original, 29-item version of the inflammatory bowel disease self-efficacy scale; IBD-SES13, the 13-item short version of the inflammatory bowel disease self-efficacy scale; ICC, intraclass correlation coefficient; SD, standard deviation; 95% CI, 95% confidence interval

**Table 3:** Main aspects of subscales and items selected for the 13-item short version of the IBD-SES

Subscales	Interpretation of main aspects	Items selected for the short version
Managing stress and emotions	Keep from stressors	1. Keep from getting stressed
	Try to alleviate negative feelings	4. Do something to reduce discouragement
	Get support from others	9. Get emotional support
Managing medical care	Keep high medication adherence	11. Take medication at instructed times
	Participate in their medical care	14. Ask doctor about illness
		15. Discuss problems with medications
Managing symptoms and disease	Manage sleep problems	19. Keep sleep problems from interfering
	Manage problems related bowel symptoms	20. Keep discomfort/pain from interfering
	Manage general symptoms	22. Keep symptoms from interfering
	Manage fatigue	24. Keep fatigue from interfering
Maintaining remission	Try to maintain remission	26. Keep disease in remission
	Engage in specific self-care	27. Engage in self-care (exercise, diet, rest)
	Engage in general self-management	29. Maintain your sense of well-being

IBD-SES, the original, 29-item version of the inflammatory bowel disease self-efficacy scale

Table 2 shows descriptive and psychometric statistics for items and subscales in the IBD-SES13. The mean score per item for each subscale was as follows: 4.6 for “managing stress and emotions”, 7.2 for “managing medical care”, 4.8 for “managing symptoms and disease”, and 5.1 for “maintaining remission.” Internal consistency analysis showed that the corrected item-total correlations were from 0.33 to 0.74, which was above the recommended value of 0.3 [21]. The Cronbach’s α of each subscale ranged from 0.68 to 0.86, which is almost within the range of the well accepted guideline of 0.7 to 0.9 [21]. The ICC (95% confidence interval) to assess test-retest reliability of each item was 0.62 (0.54–0.69) to 0.78 (0.73–0.82), which showed substantial reliability (0.6 to 0.8) [25]. Correlation with the original IBD-SES within each subscale was high (0.97–0.98).

**Fig. 2:**
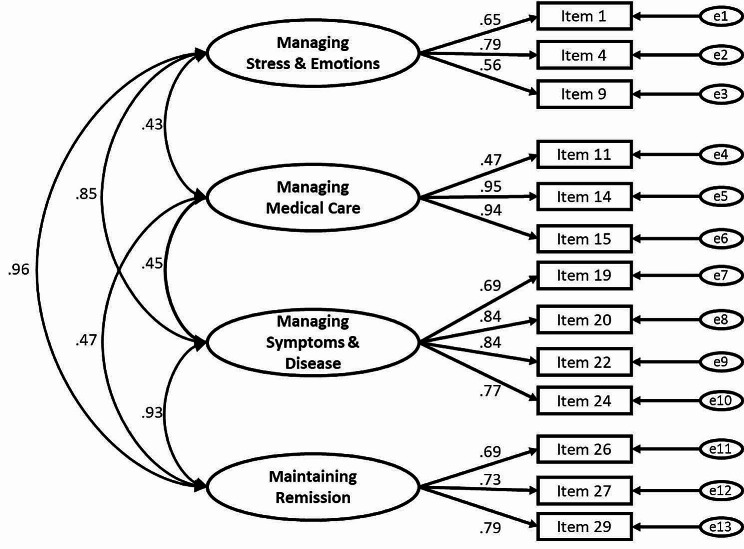
Confirmatory factor analysis of the short version of the IBD-SES The results show correlation coefficients between subscales (large ovals) and items (rectangles). The small ovals labeled e1 through e13 stand for measurement errors in each item. The sample was 873 complete data sets (no missing items). IBD-SES, the original, 29-item version of the inflammatory bowel disease self-efficacy scale

Figure 2 shows the results of confirmatory factor analysis for the IBD-SES13 based on our hypothesis. The fit indices were as follows: normed chi-square, 7.18 (*p* < 0.001); CFI, 0.94; GFI, 0.93; AGFI, 0.89; PGFI, 0.60; and RMSEA, 0.084. The chi-square test was statistically significant, but the alternate fit index indicated almost within the good or acceptable range (mean values of CFI > 0.90; GFI > 0.90; AGFI > 0.90; RMSEA < 0.08; and GFI > PGFI) [26]. 

## Discussion

This study showed that the 13-item IBD-SES13 has better psychometric properties than the original IBD-SES and demonstrated the reliability and validity of the IBD-SES13. Reduction of overlapping items may improve the usefulness of the IBD-SES in clinical settings. Overlapping items were eliminated by using a confirmatory factor analysis with sufficient sample size. The four domains of items in the original IBD-SES were retained in the IBD-SES13, and these subscales can detect specific self-management areas in which a patient needs support.

There are many PROMs in IBD, and most scales focusing on their HR-QOL or disability as well as disease activity [27]. Self-efficacy is one of the important mediators or predictors of HR-QOL [9] and measuring self-efficacy can lead strategy to promote ideal self-management behaviors. Despite the usefulness of assessing self-efficacy, few tools can access patients’ self-management, in such a situation the IBD-SES is a valuable tool. The IBD-SES has 29 items, comparable to the 32 items in the IBDQ [28] which is one of the most commonly used in clinical trials. The advantage of having many items is comprehensive coverage of the topic, increasing content validity, and deepening the data analysis. On the other hand, shortening the questionnaire is effective in increasing the response rate by decreasing their burden [18, 19], therefore, developing a shorter version is warranted. This shorter version of the IBD-SES may have advantages not only in clinical settings but also in research settings, where it may improve response rates or allow to addition of other variables and enable evaluation of more parameters to facilitate complex analyses.

There are some limitations of the present study. First, not all aspects of validity were assessed during the psychometric evaluations. Exploratory factor analysis or item response theory was not utilized in the item reduction process, as the study prioritized maintaining the same subscales as in the original version of the IBD-SES. Furthermore, sensitivity to change or minimal important difference (MID) was not explored. Understanding the sufficient magnitude of change could be beneficial. However, the results of this study can serve as reference data, given that a systematic review discovered a close concordance between mean MID and Cohen’s effect size of 0.5 [20]. It would have been ideal to perform an assessment in relation to objective or behavioral measures such as medication adherence or taking a regular cancer screening. Evaluations involving predictability of and/or sensitivity to clinical outcomes would be helpful. We have followed careful procedures regarding linguistic equivalence with the original version [17], and we believe that this shorter version is also valid, though, cross-cultural validation is also essential because of variations in local practices and norms. Additional studies are required to provide further insights into improving the usability of this scale.

## Conclusions

In conclusion, this study developed a shorter 13-item version of the IBD-SES, consisting of the same four subscales as the original scale. The results confirmed the improved validity, reliability, and psychometric properties of the IBD-SES. A shorter instrument would increase the likelihood of usage. The IBD-SES short version is suitable for clinical assessment for developing strategies to foster self-management ability.

### Electronic supplementary material

Below is the link to the electronic supplementary material.

Supplementary Material 1

## Data Availability

The data underlying this article will be shared on reasonable request to the corresponding author.

## Abbreviations

AGFI: Adjusted goodness of-fit index
CD: Crohn’s disease
CFI: Comparative fit index
GFI: Goodness-of-fit index
HR-QOL: Health-related quality of life
IBD-SES13: The 13-item short version of the inflammatory bowel disease self-efficacy scale
IBD-SES: The original, 29-item version of the inflammatory bowel disease self-efficacy scale
IBD: Inflammatory bowel disease
IBDQ: Inflammatory bowel disease questionnaire
ICC: Intraclass correlation coefficient
MID: Minimal important difference
PGFI: Parsimony goodness-of-fit index
PROMs: Patient-reported outcome measures
RMSEA: Root mean square error of approximation
SD: Standard deviation
UC: Ulcerative colitis
