# Supplementary material for: Development and validation of a 13-item short version of the inflammatory bowel disease self-efficacy scale
Source: BMC Gastroenterol. 2024 May 31;24:190. doi: 10.1186/s12876-024-03206-x (PMC11141053; doi:10.1186/s12876-024-03206-x)
Supplement: Supplementary file 1 — Supplementary Material 1 [file 12876_2024_3206_MOESM1_ESM.docx]

**Supplemental Table.** Items selected for the short version of the IBD-SES based on analysis of the original 29 items.

| **Subscales** | **Items** | **N** | **Meam±SD** | **I-T** | **ICC** | **High ^c^ correlation  with (item):** | **Removed by** | **IBD-SES13** |
| --- | --- | --- | --- | --- | --- | --- | --- | --- |
| Managing stress and emotions | **1. Keep from getting stressed** | 919 | 3.7±2.2 | 0.57 | 0.68 |  |  | **✓** |
|  | 2. Do something to reduce stress | 919 | 4.7±2.3 | 0.66 | 0.67 | 4 | Step3 |  |
|  | 3. Keep from getting discouraged | 916 | 4.4±2.3 | 0.67 | 0.71 | 4, 5, 6, 7, 8 | Step3 |  |
|  | **4. Do something to reduce discouragement** | 919 | 4.9±2.3 | 0.73 | 0.69 | 2, 3, 5, 6, 7, 8 |  | **✓** |
|  | 5. Keep from feeling sad | 919 | 4.7±2.4 | 0.72 | 0.69 | 3, 4, 6, 7, 8 | Step3 |  |
|  | 6. Do something to reduce sadness | 919 | 4.9±2.3 | 0.75 | 0.67 | 3, 4, 5, 7, 8 | Step3 |  |
|  | 7. Keep sadness/anxiety from interfering | 919 | 4.7±2.3 | 0.71 | 0.73 | 3, 4, 5, 6, 8 | Step3 |  |
|  | 8. Do something to reduce interference of sadness/anxiety | 919 | 4.9±2.3 | 0.73 | 0.73 | 3, 4, 5, 6, 7 | Step3 |  |
|  | **9. Get emotional support** | 919 | 5.1±2.5 | 0.54 | 0.74 |  |  | **✓** |
| Managing medical care | 10. Follow medication prescription | 916 | 7.7±2.5 Ceiling ^a^ | 0.38 | 0.64 | 11, 12 | Step1 |  |
|  | **11. Take medication at instructed times** | 916 | 7.0±2.6 | 0.41 | 0.69 | 10, 12 |  | **✓** |
|  | 12. Take medication as directed to prevent flare-up | 911 | 7.6±2.5 Ceiling ^a^ | 0.44 | 0.68 | 10, 11 | Step1 |  |
|  | 13. Work with providers on treatment plan | 919 | 7.6±2.3 | 0.57 | 0.66 | 14, 15 | Step3 |  |
|  | **14. Ask doctor about illness** | 918 | 7.3±2.3 | 0.62 | 0.69 | 13, 15, 16, 17 |  | **✓** |
|  | **15. Discuss problems with medications** | 918 | 7.2±2.4 | 0.63 | 0.69 | 13, 14, 16, 17 |  | **✓** |
|  | 16. Work out differences with doctors | 918 | 6.7±2.5 | 0.62 | 0.66 | 14, 15, 17 | Step3 |  |
|  | 17. Ask doctor about medications | 916 | 7.1±2.4 | 0.59 | 0.69 | 14, 15, 16 | Step3 |  |
| Managing symptoms and disease | 18. Reduce symptoms | 914 | 5.8±2.3 | 0.69 | 0.59 Low ^b^ |  | Step2 |  |
|  | **19. Keep sleep problems from interfering** | 916 | 5.4±2.6 | 0.63 | 0.64 |  |  | **✓** |
|  | **20. Keep discomfort/pain from interfering** | 917 | 5.0±2.2 | 0.72 | 0.62 |  |  | **✓** |
|  | 21. Keep diarrhea/urgency from interfering | 917 | 4.6±2.2 | 0.62 | 0.59 Low ^b^ | 22 | Step2 |  |
|  | **22. Keep symptoms from interfering** | 914 | 4.8±2.2 | 0.73 | 0.66 | 21 |  | **✓** |
|  | 23. Decrease fatigue | 917 | 4.3±2.1 | 0.67 | 0.67 | 24 | Step3 |  |
|  | **24. Keep fatigue from interfering** | 915 | 4.3±2.1 | 0.70 | 0.67 | 23 |  | **✓** |
| Maintaining remission | 25. Manage your disease | 919 | 5.3±2.1 | 0.74 | 0.70 | 26 | Step3 |  |
|  | **26. Keep disease in remission** | 913 | 5.3±2.2 | 0.64 | 0.69 | 25 |  | **✓** |
|  | **27. Engage in self-care (exercise, diet, rest)** | 919 | 5.3±2.2 | 0.68 | 0.65 |  |  | **✓** |
|  | 28. Engage in stress management program | 904 | 3.6±2.0 | 0.49 | 0.56 Low ^b^ |  | Step2 |  |
|  | **29. Maintain your sense of well-being** | 919 | 4.9±2.3 | 0.72 | 0.78 |  |  | **✓** |

The 13 items retained in the short version are in bold.
^a^ Ceiling effect was defined as the mean + SD exceeding the scale’s range;
^b^ Low ICC was defined as an ICC value of <0.6; ^c^ High correlation between an item pair was defined as a correlation coefficient of ≥0.7;
IBD-SES13, the 13-item short version of the inflammatory bowel disease self-efficacy scale; ICC, intraclass correlation coefficient; I-T, Corrected Item-Total correlation coefficient; SD, standard deviation.
